# The effects of different types of RAGT on balance function in stroke patients with low levels of independent walking in a convalescent rehabilitation hospital

**DOI:** 10.1515/med-2025-1212

**Published:** 2025-06-27

**Authors:** Dae-Hwan Lee, Bong-sik Woo, Jong-hyeon Lim, Jin-ook Choi, Yong-Hwa Park

**Affiliations:** Rehabilitation, Immanuel Medical Rehabilitation Hospital, Cheongju-si, Republic of Korea; Medical, Immanuel Medical Rehabilitation Hospital, Cheongju-si, Republic of Korea

**Keywords:** stroke, FAC, BBS, FMA-LE, rehabilitation, balance, RAGT, convalescent rehabilitation hospital

## Abstract

**Background and aim:**

Stroke patients with low levels of walking independence often experience persistent deficits in gait and balance, which significantly limit their functional mobility and quality of life. Robotic-assist gait training (RAGT) has emerged as a promising intervention to promote motor recovery and improve postural control in this patients. While previous studies have demonstrated the benefits of RAGT, few have directly compared the effects of fixed end-effector type and mobile robotic gait devices in patients with severely impaired ambulation. This study aimed to investigate and compare the effects of these two robotic gait training on balance and lower extremities motor recovery in stroke patients classified as functional ambulation category 0 to 2.

**Methods:**

Twenty-eight stroke patients were randomly assigned to either end-effector or mobile robot groups, undergoing 12 weeks of therapy with one daily robotic session and seven conventional physical therapy sessions per week. Outcomes were measured using the Berg Balance Scale (BBS) and Fugl–Meyer Assessment for Lower Extremity (FMA-LE), with subcategory analysis for reflex activities, volitional movement within synergies, volitional movement mixing synergies, volitional movement with little or no synergy, normal reflex activity, and coordination/speed.

**Results:**

Both groups showed significant improvements in BBS and FMA-LE, with the mobile robot group showing greater gains. Both groups improved in walking independence, though no significant difference was found between them. Subcategory analysis showed improvements in reflex activities and coordination/speed in both groups, but volitional movement within synergies and volitional movement with little or no synergy improved only in the mobile robot group. Correlation analysis revealed significant relationships between FAC and BBS, and BBS and reflex activities. Volitional movement within synergies and volitional movement mixing synergies had high correlations with motor recovery.

**Conclusion:**

Both robotic methods effectively improved balance and motor recovery, with mobile robots showing greater potential for enhancing functional autonomy.

## 1 Introduction

Paralysis of the lower limbs in stroke patients can cause challenges in mobility, balance and lower limbs motor functions. Lower limb paralysis usually presents as weakness, sensory loss, spasticity, or paralysis in one or both legs, and these symptoms make it difficult for patients to perform daily movements and maintain balance [1]. A stroke impacts the brain’s ability to plan and execute movements. This is particularly crucial for dynamic activities like walking, which require coordinated activity of various muscle groups and balance maintenance [2]. Difficulties in movement planning and execution can lead to instability while walking and increase the risk of falls. Due to weakness and sensory loss in the lower limbs, stroke patients find it challenging to maintain balance while standing. The risk of stumbling or falling increases, especially when shifting weight to one side or changing posture. Additionally, if foot and ankle functions are impaired, it becomes harder to adapt to changes in the ground surface, making it even more difficult to maintain balance [3]. When lower limb paralysis occurs, the patient’s gait pattern can become abnormal because of abnormal lower motor function. Common issues include foot drop, where the patient cannot lift the foot properly, leading to dragging toes on uneven surfaces, and difficulty fully bending one leg, which may cause the foot to catch on the ground. These problems slow down walking speed, create irregular gait patterns, and increase the risk of falling [4]. Moreover, spasticity resulting from a stroke causes excessive contraction of certain leg muscles, making the leg stiff and hindering the flexibility of the knee and ankle during walking. Lower limb paralysis often prevents patients from properly supporting their body weight on one leg, which makes it challenging to maintain balance while walking. As a result, many patients need to use assistive devices such as canes or walkers or require help from others when walking. To compensate for muscle weakness and spasticity during walking, patients often adopt irregular or abnormal gait patterns, leading to increased energy consumption [5]. Sensory impairments caused by stroke may involve alterations in cutaneous sensation, muscle and joint proprioception, and kinesthesia, leading to a diminished ability to accurately perceive body position and movement. These sensory deficits compromise postural control during gait and delay responses to unexpected perturbations, thereby increasing the risk of falls. Gait instability is further exacerbated in environments with uneven surfaces or obstacles. As a result, patients expend excessive concentration and energy even during short-distance ambulation, often experiencing early fatigue. These challenges hinder the performance of essential daily activities, such as going out independently, using public transportation, climbing stairs, bathing, cooking, and house cleaning [6]. These issues can be improved through rehabilitation and physical therapy, including lower limb strengthening exercises, balance training, and gait training. If these problems are not properly managed and treated in the early stages, long-term complications such as joint contractures, muscle weakness, and chronic pain may develop [3,4,5,6].

In line with Article 18 of the Act on the Rights of Persons with Disabilities to Health, the Korean government implemented the Convalescent Rehabilitation Medical Institution System. Launched as a pilot program in October 2017 across 15 hospitals, this system establishes a comprehensive rehabilitation structure in which physical, occupational, and speech therapists deliver up to 16 units (15 min per unit) of therapy per day. It targets neurological conditions such as stroke, brain injuries, and spinal cord injuries. Both Japan and Korea provide an inpatient rehabilitation period of up to 180 days for patients with central nervous system disorders. In Japan, the system allows for intensive rehabilitation of up to nine units per day, with each unit consisting of 20 min. By extending hospitalization periods up to 6 months for patients in need of intensive rehabilitation, this system aims to reduce long-term disabilities and support patients’ reintegration into society [7,8,9]. For patients who have passed the acute phase of stroke and whose vital signs have stabilized, early therapeutic intervention is crucial, as it allows for functional recovery and preparation for independent living [10]. Intensive treatment focuses on maximizing physical recovery, with particular emphasis on motor functions of the upper and lower limbs, balance, and gait training. In these hospitals, physical therapists, occupational therapists, rehabilitation specialists, and speech therapists work together to develop individualized treatment plans tailored to each patient’s condition. This team approach supports the patient’s overall recovery, providing physical, psychological, and social rehabilitation simultaneously [7,8,9,11]. Early rehabilitation promotes neuroplasticity, compensating for damaged brain functions and increasing the likelihood of recovering lost physical functions. Early rehabilitation following stroke is effective because it takes advantage of the brain’s heightened neuroplasticity during the early subacute phase. Intensive task-oriented training at this stage promotes reorganization of neural circuits, prevents disuse atrophy, and enhances functional recovery through use-dependent learning. Furthermore, early mobilization reduces the risk of secondary complications and boosts patient motivation for active participation in recovery [9,12]. Physical therapy help strengthen muscles and restore flexibility. This is particularly important for recovering muscle strength in paralyzed lower limbs and increasing the range of motion in joints [13]. Gait and balance training enhance the patient’s ability to move independently, reducing the risk of falls and enabling them to perform daily activities. Through occupational therapy, patients receive training in activities of daily living, such as washing, dressing, and eating. This prepares them for independent living after discharge and contributes to an improved quality of life [14]. Patients receive psychological support during their treatment and experience social rehabilitation by interacting with others who share similar experiences. This process helps stabilize the patient’s emotional state and increases their motivation for rehabilitation [15]. In conclusion, intensive rehabilitation therapy at convalescent rehabilitation hospitals plays a crucial role in promoting functional recovery, preparing stroke patients for independent living, and enhancing their overall quality of life. The Republic of Korea’s convalescent rehabilitation hospitals have been implemented by benchmarking Japan’s convalescent rehabilitation hospital model. The Japan’s current convalescent rehabilitation hospitals play a crucial role in maximizing functional recovery through early intervention and intensive rehabilitation therapy, helping patients prepare for independent living. A key aspect of Japan’s convalescent rehabilitation hospitals is the establishment of personalized treatment plans tailored to each patient’s condition and needs, realized through multidisciplinary team collaboration. The smooth coordination between acute care hospitals and convalescent rehabilitation hospitals ensures that patients receive rehabilitation treatment in a timely manner. This system also supports ongoing rehabilitation and social reintegration post-discharge. Japan provides legal and financial support to convalescent rehabilitation hospitals, subsidizing rehabilitation treatment costs through the national health insurance system, allowing patients to receive rehabilitation care without financial burden [7,8,9,16,17,18].

Effective rehabilitation is crucial for stroke function recovery, focusing on restoring function and improving quality of life. Balance and lower limb impairments increase dependency and the risk of falls, necessitating targeted rehabilitation efforts to restore mobility and independence [19]. Target muscle strength enhancement, particularly in the lower limbs, is essential for stable gait and balance. Techniques include resistance exercises and isometric training, which have been proven to significantly improve muscle power and directly enhance balance and walking ability [20]. It focuses on improving both static and dynamic balance skills to prevent falls. Techniques involve activities like standing on one leg, using balance boards, and practicing slow movement like Tai chi [21]. Evidence shows that balance training significantly enhances the ability to maintain and regain balance during movement, improving daily activity performance. It aims to enhance walking ability and efficiency through treadmills, over-ground walking exercises, and robotic assistive devices. These methods facilitate repetitive practice and help correct abnormal gait patterns, with controlled trials showing improvements in speed, endurance, and symmetry in gait post-stroke [22]. In this study, we aimed to confirm the effect of robot training on improving the balance and motor and sensory recovery of stroke patients. Robotic-assist gait training (RAGT) can be broadly divided into two categories: end-effector and mobile robot.

In recent years, the field of stroke rehabilitation has undergone significant changes with the emergence of RAGT, bringing about a paradigm shift that addresses the limitations of traditional rehabilitation methods. Various studies have demonstrated the effectiveness of RAGT, with reports suggesting that it is particularly more effective in acute stroke patients than in those with chronic stroke [22,23]. Additionally, RAGT in the acute phase of stroke patients who are fully dependent has been reported to improve maximal aerobic capacity, maximal heart rate, and exercise tolerance [24].

RAGT utilizes advanced robotic devices and systems carefully designed to support, strengthen, and guide the lower limbs during gait. Mobile robot gait training is a rehabilitation robot system designed to help patients with stroke or other motor impairments recover their walking ability. This system is typically worn around the legs and waist, assisting with walking movements while allowing spatial movement [23,24,25]. The primary goal of mobile robots is to support or guide the patient’s movements while promoting a natural walking pattern. Most mobile robots are equipped with a harness system, is designed to prevent falls, and typically employs a pelvic-type harness. It is secured to the upper part of the robotic device and provides vertical body weight support during gait training [26,27]. While exoskeletons and end-effector devices are fixed type, mobile systems differ in that they provide body weight support through a harness, allowing the robot to assist the patient’s walking while moving along with the patient through a corridor [28]. Mobile robotic gait training offers a distinct advantage over stationary fixed-type devices by allowing spatial movement during training. Unlike fixed systems that limit movement to a predetermined path, mobile robots support overground walking and enable patients to practice gait in environments that more closely resemble real-world conditions. These systems not only retrain functional gait patterns but also facilitate a variety of multidirectional movements, such as trunk rotation, flexion, extension, and lateral bending, which are commonly used in daily life. By exposing patients to dynamic and unpredictable training contexts, mobile RAGT enhances adaptability to varying environments and promotes greater postural control and motor responsiveness, thereby supporting a more functional and transferable recovery process [26,28,29].

In this study, we aim to investigate the effects of gait training using fixed-type devices as end-effector and mobile systems on patients with low levels of independent walking ability. Previous studies have reported that mobile gait training is effective in improving walking speed, step count per minute, and walking duration [30]. Ten-week training programs have shown improvements in timed up and go test (TUG), BBS, and dynamic gait index scores [31]. Additionally, most studies, except for a few, have reported improvements in 10 m walk test, FAC, BBS, MBI, and TUG scores following end-effector training [31,32,33].

The patient’s level of walking independence can be assessed using the FAC. FAC is a scale that evaluates a patient’s walking ability and categorizes how independently they can walk on a scale from 0 to 5. The FAC assessment has a high level of reliability, with an inter-rater reliability of 0.982 and an intra-rater reliability of 0.991. In this study, we compared the effects of gait training using end-effector and mobile robots on patients with low levels of walking independence (FAC scores of 0–2). FAC scores of 0–2 indicate an inability to walk or severely limited ambulation, while scores of 3 or higher reflect a level of supervision or independent walking ability. Therefore, an improvement in FAC score can serve as an indicator of the ability to perform daily activities without assistance, and is closely associated with successful rehabilitation outcomes, early discharge, and reintegration into the community [34,35]. Additionally, we compared the impact on sensory and motor recovery in patients who are unable to walk independently but have a high level of balance ability (patients with high BBS scores).

The Fugl–Meyer assessment (FMA-LE) specifically evaluates motor function, balance, and joint movement in the lower extremities of stroke patients. It uses a 3-point scale (0 = cannot perform, 1 = partially performs, 2 = fully performs) to score tasks such as flexion, extension, and reflex activities of the hip, knee, and ankle. The total score reflects the patient’s level of motor recovery, with higher scores indicating greater functional ability and better control of the lower extremities, which aids in developing personalized rehabilitation plans [36]. Although previous studies have compared recovery in sensory and motor function using the total FMA-LE score, this study aims to compare the individual subcomponents of the FMA-LE.

Previous research on RAGT typically involved therapeutic interventions lasting 4–10 weeks. Studies show that some improvements are generally observed by the 4th week, with significant effects occurring between the 6th and 8th weeks. Some studies report that the effects of interventions between 6 and 10 weeks may last up to 5–6 months, but most research emphasizes the necessity of continued management and follow-up rehabilitation to maintain these therapeutic effects [22,23,24,25,26,28,29,31,32,37].

To date, many studies on fixed- and mobile-type robots have been reported, and although some show mixed results, most researchers agree on their effectiveness in restoring motor functions in patients. However, there is a lack of research on the effects of mobile robots and end-effector devices. Therefore, this study aims to investigate the effects of RAGT using mobile systems and end-effector devices on balance and motor recovery in stroke patients with low levels of independent walking. Furthermore, this study compares the correlations between the subcomponents of the FMA-LE and their impact on balance, examining how each subcomponent influences balance. It is hypothesized that the effectiveness of training will differ based on the level of the FMA-LE subcomponents in patients with low independent walking ability.

## 2 Materials and methods

### 2.1 Subjects

For this experiment, outpatients from I Hospital in C City, Korea, were recruited. The inclusion criteria were (1) patients who had developed a stroke for more than 1 month and less than 6 months, (2) FAC 0–2, (3) hemiplegic patients without adjust devices classified as Brunnstrom’s motor recovery stage, (4) ability to understand and follow oral instructions, (5) Patients without orthopedic disease in the lower extremity, and (7) Mini-Mental State Examination, Korean version (MMSE-K) score of 20 or higher. The exclusion criteria were (1) visual, auditory, or vestibular disorders, and (2) functional problems of the lower limb due to other neurological problems unrelated to stroke.

**Figure 1:**
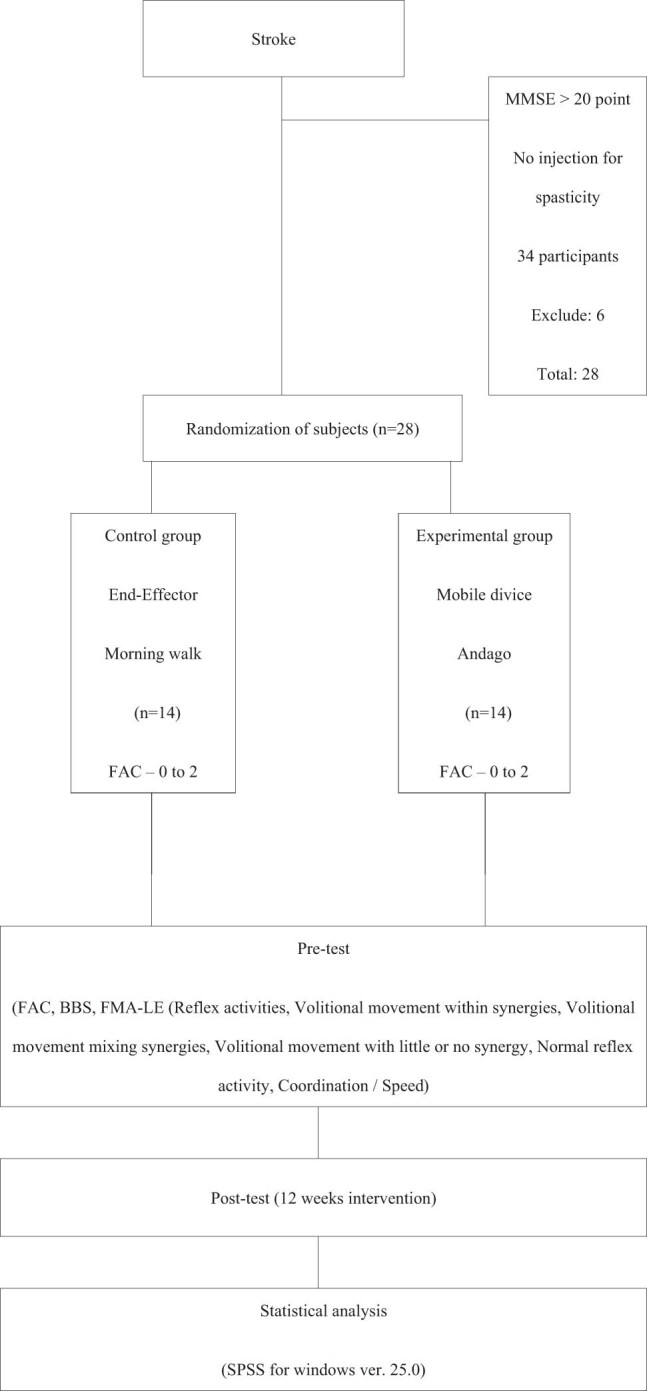
Flow chart.

A total of 34 patients were initially screened for eligibility. After applying inclusion and exclusion criteria, 28 participants were enrolled and randomly assigned to two groups: 14 to the fixed-type robotic gait training group and 14 to the mobile-type robotic gait training group. All participants completed the 12-week intervention without dropout and were included in the final analysis (Figure 1). The average age was 67.32 ± 13.07 years, the average height was 163.78 ± 8.64 cm, and the average weight was 61.96 ± 7.85 kg. The average MMSE-K score was 23.42 ± 2.50. Stoke type was a seizure type of all patients, and the average time elapsed after stroke was 29.10 ± 17.92 days. All research procedures were performed under the supervision of the Institutional Review Board in accordance with the Declaration of Helsinki. All experimental procedures and protocols have been approved by the research ethics committee following the guidelines of Cheongju University (IRB-1041107-202404-HR-010-01).

### 2.2 Design

This study was conducted as a randomized clinical trial, with the sample selected through systematic sampling, and the trial was carried out using a single-blind test. The study subjects were randomly assigned into two groups. The control group performed gait training using a fixed end-effector-type robotic device, the Morning Walk S200 (MW-S200, Curexo, Seoul, Korea), which provides stationary gait training in a fixed location. The experimental group conducted gait training using the Andago^®^ V2.0 (Hocoma AG, Volketswil, Switzerland), a mobile robotic gait device that allows the patient to walk through corridors while receiving body weight support. *A priori* power analysis using G*power indicated that a minimum of 102 participants would be required to detect a medium effect size (Cohen’s *d* = 0.5) with a significance level of 0.05 and a power of 0.80. However, due to clinical limitations, only 28 participants were recruited and completed the study. Accordingly, this study may be considered an exploratory or pilot trials. All participants completed this study without dropout.

### 2.3 Intervention

The patients underwent rehabilitation therapy 5 times a week, with a total of 16 sessions of rehabilitation training per day. Two sessions were conducted using different robotic devices for intervention, while the remaining 14 sessions followed the same training protocol comprehensive rehabilitation. Each session lasted for 15 min, during which the walking speed was gradually increased from 0.2 to 1.2 m/s, with increments every 5 min. The therapists were unaware of the study’s objectives during the intervention.

The control group used the end-effector robot as Morning walk S200 (MW-S200, Curexo, Seoul, Korea). The Morning Walk S200 is a robotic automated system designed for muscle rebuilding and joint mobility recovery to aid in the restoration of walking ability. It monitors the patient’s condition by measuring blood SpO_2_ and heart rate using an optical detection system through an oxygen saturation probe (sensor). The unique seated weight support system of Morning Walk minimizes the time required for patient boarding and therapy preparation (within 3 min), allowing for quick and efficient training. It offers a boarding/dismounting mode for severely impaired patients and can simulate natural inverse pendulum trunk movement with its saddle-shaped weight support, accommodating patients up to 200 kg. The system can replicate walking on flat surfaces, ascending and descending stairs, and navigating inclines, with step adjustments for stairs in three stages (7, 12 and 17 cm) and ramps in four stages (5°, 10°, 15°, and 20°). The independent walking pattern configuration with separate left and right footplates allows for effective, gradual therapy. The Virtual Reality software enhances the therapeutic experience, offering high treatment efficacy for rehabilitation. Active patient participation is encouraged through the variable speed mode, which adjusts walking speed based on the pressure applied to the footplates, and the variable trajectory mode, which alters footplate movement based on the force used to lift them. In this study, only speed variations were used for the intervention, without adjusting the incline. In this study, the treatment was administered by an experienced physical therapist with 5 years of clinical experience.

The experimental group used the mobile robot as Andago^®^ V2.0 (Hocoma AG, Volketswil, Switzerland). Andago^®^ V2.0 is a therapeutic tool designed to allow physical therapists and other clinicians to safely perform overground gait and balance training for patients with walking or balance impairments. It offers a dynamic, individually adjustable weight support system that facilitates appropriate lateral weight shifting, supports body weight against gravity, and protects patients from falling while standing or walking. Andago^®^ V2.0 can be used for a variety of overground gait and balance training modes, including standing, walking in a straight line, self-initiated walking along freely chosen paths, and clinician-controlled walking along predetermined routes. By supporting the patient’s weight and posture, Andago^®^ V2.0 eliminates the need for the clinician to physically bear the patient’s weight, thus facilitating overground gait and balance training. It should always be used under the supervision of a qualified clinician who has read and understood the user manual. In this study, the treatment was administered by an experienced physical therapist with 5 years of clinical experience. During Andago training, only speed variations were applied, and the training was conducted on flat ground.

### 2.4 Outcome measure

#### 2.4.1 FAC

FAC is a simple tool used to assess a patient’s walking ability, particularly after neurological damage such as a stroke. It evaluates how independently a patient can ambulate, based on the clinician’s observation of their walking performance.

The FAC is divided into six levels, with each level indicating the amount of assistance the patient requires for walking:

FAC 0: Non-functional walking. The patient cannot walk under any circumstances.

FAC 1: Ambulation dependent on assistance (requiring support). The patient requires continuous support from two or more people to walk and cannot move independently.

FAC 2: Ambulation with maximum assistance (physical assistance required). The patient requires continuous physical assistance from one person to walk.

FAC 3: Ambulation with minimal assistance (light support needed). The patient cannot walk independently in everyday environments but only needs light physical contact or support for balance.

FAC 4: Ambulation with supervision (independent with supervision). The patient can walk independently without physical help, but requires supervision or standby assistance to ensure safety or prevent falls.

FAC 5: Independent walking. The patient can walk independently in all environments without any assistance or supervision.

The FAC provides a straightforward and effective way to assess a patient’s walking ability, helping to monitor their progress and guide rehabilitation plans, particularly in terms of independence in mobility. In this study, patients with FAC scores of 0–2 were selected to compare those with low levels of independence.

#### 2.4.2 Berg balance test (BBS)

BBS is a clinical tool used to assess a person’s balance and risk of falling, particularly in elderly individuals or those with balance impairments due to neurological conditions, stroke, or injury. The test consists of 14 simple tasks that evaluate static and dynamic balance abilities in a controlled, measurable way.

Each task in the BBS is scored on a scale from 0 to 4, with 0 indicating inability to perform the task and 4 representing full independence. The maximum total score is 56. Tasks include activities like sitting to standing, standing unsupported, transferring between chairs, standing with eyes closed, turning to look behind, and placing one foot in front of the other.

A higher score generally indicates better balance and a lower risk of falling, while a lower score suggests greater impairment. It is widely used in clinical settings to monitor progress, guide treatment plans, and predict fall risk. Clinicians often use the BBT in combination with other assessments to get a comprehensive view of a patient’s mobility and balance.

#### 2.4.3 FMA-LE

The FMA-LE consists of six subcategories that evaluate various aspects of lower extremity function, particularly after a stroke. Each subcategory focuses on a different aspect of motor control and reflex activity. The total score for FMA-LE is 34 points. This score evaluates lower limb motor function, balance, and joint movement, with higher scores indicating greater functional recovery.

#### 2.4.4 Reflex activities

This category assesses basic reflex responses in the lower extremities. It typically involves testing knee and ankle reflexes to check whether they are normal, hyperactive, or absent.

#### 2.4.5 Volitional movement within synergies (synergies)

This evaluates the patient’s ability to perform volitional movements within specific movement patterns. The assessment checks how well the patient can execute flexion and extension patterns, such as bending the knee or lifting the ankle.

#### 2.4.6 Volitional movement mixing synergies (mixed synergies)

This subcategory assesses the ability to perform volitional movements that combine multiple synergy patterns. It looks at how effectively the patient can control complex, coordinated movements involving different muscle groups in the lower extremity.

#### 2.4.7 Volitional movement with little or no synergy (volitional movement)

This evaluates the patient’s ability to independently move specific muscles or joints without relying on synergy patterns. It focuses on isolated movements, such as the ability to move the ankle freely without coordinating it with other movements.

#### 2.4.8 Normal reflex activity (normal reflex)

This subcategory checks for the presence of normal reflexes in the lower extremities. The goal is to assess whether the normal reflex patterns and muscle responses are present, reflecting the overall condition of the nervous system.

#### 2.4.9 Coordination/speed

This evaluates the smoothness and speed of volitional movements in the lower extremities. The patient’s coordination and speed are tested to see how quickly and accurately they can perform tasks, highlighting both motor control and functional ability.

### 2.5 Statistical analysis

In this study, IBM SPSS Statistics 25.0 was used for statistical analysis. The mean and standard deviation of each variable measured to evaluate each item were calculated, and normality was assessed using the Kolmogorov–Smirnov test. The BBS and FMA-LE scores satisfied the assumption of normality, whereas the FAC and sub-items of the FMA-LE did not. An independent *t*-test was used to compare between-group differences, and a paired *t*-test was used to assess within-group differences for ratio scale variables (BBS, FMA-LE). For ordinal scale variables (FAC and FMA-LE sub-items), the Mann–Whitney *U* test and Wilcoxon signed-rank test were applied. Correlation analysis was performed to examine relationships among variables, and regression analysis was conducted to identify factors associated with FAC. Spearman’s rank correlation analysis was conducted to examine the relationships among all variables. In addition, multiple regression analysis was performed to identify the factors influencing the BBS. Statistical significance was set at *p* < 0.05.

## 3 Results

The characteristics of the subjects are shown in Table 1, and there were no statistically significant differences between the two groups.

**Table 1:** General characteristics of the subjects

Group		Control		Experimental		*p*
Age-old		69.14	13.64	69.50	12.98	0.944
Height		163.42	10.02	164.14	7.36	0.832
Weight		61.57	7.7	62.35	8.27	0.797
Sex (*n*)		Male (9), female (5)		Male (8), female (6)		0.712
Onset (days)		26.21	20.43	32.00	15.22	0.403
Stroke type	Hemorrhage	4		6		0.449
	Ischemic	10		8		
Affected side	Right side	7		6		0.717
	Left side	7		8		
MMSE-K		23.57	2.84	23.28	2.19	0.769
FAC		1.07	0.82	1.14	0.770	0.825

*SD: standard deviation.

*p < 0.05*, *p* < 0.01**, *p* < 0.001*** (intergroup).

*p < 0.05a, < 0.01b, < 0.001c (intragroup).

Control: end-effector robotic training group; experimental: mobile robotic training group.

The BBS results are presented in Table 2, and statistically significant improvements were observed in both groups; however, no significant differences were found between the groups.

**Table 2:** Comparison of BBS

BBS	Control	Experimental	*t*	*p*
Pre-test	18.57 ± 11.50	24.57 ± 10.44	−1.445	0.160
Post-test	23.50 ± 9.92	33.64 ± 11.08	−2.550	0.017*
*t*	3.443	5.281		
*p*	0.004**	0.000***		

*p < 0.05*, *p* < 0.01**, *p* < 0.001***.

Control: end-effector robotic training group; experimental: mobile robotic training group; BBS: Berg balance scale.

FMA-LE results are presented in Table 3, and statistically significant improvements were observed in both groups; however, no significant differences were found between the groups.

**Table 3:** Comparison of FMA-LE

FMA-LE	Control	Experimental	*t*	*p*
Pre-test	19.08 ± 7.92	15.50 ± 5.31	1.400	0.173
Post-test	21.57 ± 5.60	22.57 ± 5.35	−0.444	0.660
*t*	2.917	9.462		
*P*	0.12*	0.000***		

*p < 0.05*, *p* < 0.001***.

Control: end-effector robotic training group; experimental: mobile robotic training group; FMA-LE: Fugl-Meyer assessment lower extremities.

In the Mann–Whitney and Wilcoxon tests, both groups showed statistically significant differences before and after treatment for FAC, reflex activities, and coordination/speed. However, for synergies, mixed synergies, and volitional movement, statistically significant improvements were observed only in the experimental group (Table 4).

**Table 4:** Mann–Whitney test and Wilcoxon test

	Group	Mean	SD	*U*	*P* (intragroup)
FAC pre	Control	1.07	0.82	93.50	0.825
	Experimental	1.14	0.77		
FAC post	Control	1.93	0.94**	95.50	0.901
	Experimental	1.86	11.50**		
MR pre	Control	2.07	1.20	66.50	0.097
	Experimental	1.86	0.53		
MR post	Control	2.57	1.65**	80.00	0.352
	Experimental	3.14	1.29**		
Syner pre	Control	9.79	4.20	84.00	0.516
	Experimental	9.36	3.45		
Syner post	Control	10.71	2.99	85.50	0.559
	Experimental	11.07	3.47**		
Mix syner pre	Control	2.43	1.50	72.50	0.219
	Experimental	1.86	0.77		
Mix syner post	Control	2.71	1.26	95.50	0.904
	Experimental	2.64	1.36**		
Volitional pre	Control	2.00	1.46	65.00	0.111
	Experimental	1.14	1.29		
Volitional post	Control	2.00	1.46	81.00	0.419
	Experimental	2.43	0.93**		
NR pre	Control	—	—	—	—
	Experimental	—	—		
NR post	Control	—	—	—	—
	Experimental	—	—		
C/S pre	Control	2.79	1.76	47.50a	0.017a
	Experimental	1.21	1.31		
C/S post	Control	3.64	1.73*	86.00	0.575
	Experimental	3.29	1.77**		

*SD: standard deviation.

*p < 0.05*, *p* < 0.01** (intergroup), *p < 0.05a, < 0.01b, < 0.001c (intragroup).

Control: end-effector robotic training group; experimental: mobile robotic training group.

FAC: functional ambulation category; MR: reflex activities; Syner: volitional movement within synergies; Mix syner: volitional movement mixing synergies; Volitional: volitional movement with little or no synergy; NR: normal reflex activity; C/S: coordination/speed.

Through correlation analysis, FAC showed a correlation of 0.403 with BBS, which was statistically significant. BBS exhibited a correlation of 0.470 with reflex activities, and this was also statistically significant. The FMA-LE subcategories, excluding normal reflex, showed statistically significant correlations. Specifically, synergy had a high correlation of 0.821, mixed synergies had 0.761, reflex activities had 0.528, volitional movement had 0.530, and coordination/speed showed a moderate correlation of 0.583. Reflex activities demonstrated a moderate correlation of 0.426 with synergy, which was statistically significant. Synergy had a moderate correlation of 0.567 with mixed synergies and 0.397 with volitional movement, both of which were statistically significant. Mixed synergies had a moderate correlation of 0.553 with coordination/speed, which was also statistically significant (Table 5).

**Table 5:** Correlation analysis of each elements

	Onset	FAC	BBS	FMA-LE	MR	Syner	Mix syner	Volitional	NR	C/S
Onset	1.000									
FAC	−0.133	1.000								
BBS	−0.260	0.403*	1.000							
FMA-LE	0.331	0.760	0.278	1.000						
MR	0.061	0.254	0.470*	0.528**	1.000					
Syner	0.339	0.112	0.044	0.821**	0.426*	1.000				
Mix syner	0.281	−0.140	0.139	0.761**	0.207	0.567**	1.000			
Volitional	0.177	−0.092	0.083	0.530**	0.318	0.397*	0.223	1.000		
NR	—	—	—	—	—	—	—	—	—	—
C/S	0.039	−0.045	0.274	0.583**	0.195	0.195	0.553**	0.069	—	1.000

*SD: standard deviation.

*p < 0.05, *p* < 0.01**.

FAC: functional ambulation category; BBS: Berg balance scale; FMA-LE: Fugl*–*Meyer assessment lower extremities; MR: reflex activities; Syner: volitional movement within synergies; Mix syner: volitional movement mixing synergies; Volitional: volitional movement with little or no synergy; NR: normal reflex activity; C/S: coordination/speed.

In the regression analysis, a constant of 4.05 was observed, and for each change in FAC, the BBS score increased by 2.616 points, which was statistically significant. Additionally, the variance inflation factor (VIF) was 1.164, indicating no significant multicollinearity. The R-squared value was 0.482. No other variables showed statistical significance (Table 6).

**Table 6:** Regression analysis of each element for BBS

Independent variable	*B*	*β*	*t*	TOL	VIF
Constant	4.05		0.041		
Onset	−0.4	−0.67	−299	0.723	1.384
FAC	7.017	2.616	2.682*	0.859	1.164
MR	0.089	1.460	0.061	0.775	1.291
Syner	1.461	0.404	1.754	0.449	1.994
Mix syner	−0.945	−0.105	−0.425	0.449	2.227
Volitional	0.316	0.034	0.161	0.627	1.596
C/S	0.665	0.100	0.399	0.435	2.296

*p < 0.05*, < 0.01**, *p* < 0.001***.

FAC: functional ambulation category; BBS: Berg balance scale; MR: reflex activities; Syner: volitional movement within synergies; Mix syner: volitional movement mixing synergies; volitional: volitional movement with little or no synergy; C/S: coordination/Speed; TOL: tolerance; VIF: variance inflation factor.

## 4 Discussion

This study investigated the effects of fixed end-effector type and mobile robotic gait device approaches on balance and motor function recovery in stroke patients with non-independent walking levels. While the positive effects of robotic rehabilitation have been well established, this study was conducted to address the lack of research on the specific impact of each method on patients with non-independent walking levels, comparing the correlations of FMA-LE subcomponents on balance, and suggesting the most effective approach for improving balance ability. Considering the functional differences between patients with low and high levels of independent walking, this study provides foundational data for personalized rehabilitation plans by comparing the effects of various training methods [11]. Since walking recovery is directly linked to the quality of life for stroke patients, it is essential to maximize functional recovery through targeted training [23]. Additionally, this study aims to determine the most effective robotic treatment for patients with low levels of independent walking in the subacute phase, enabling a more systematic and efficient approach to rehabilitation.

In this study, both groups underwent comprehensive rehabilitation five times a week, with 14 sessions per day, for a total of 12 weeks. Additionally, the effects of the two groups were compared based on the difference in robot types, with one group using a mobile robot and the other using a fixed type robot for two sessions per day. The results showed that the group using the mobile robot demonstrated greater improvements in the BBS and FMA-LE scores compared to the group using the fixed-type robot. This suggests that the mobile robot may be more effective in retraining natural gait patterns by providing fixed-type robot gait training [38]. The mobile robot allows free movement in real-world environments, likely contributing to functional recovery by engaging multiple muscles and joints simultaneously [27,38].

On the other hand, there was no statistically significant difference between the two groups in terms of FAC scores but both groups showed improved walking independence after the treatment. This indicates that both mobile- and fixed-type robots are effective for gait training, highlighting the importance of selecting appropriate training methods tailored to the patient’s individual condition and needs [34,39].

While the fixed-type robot may be effective in repeatedly stimulating and strengthening specific joints and muscles, the mobile robot offers more complex movement patterns and gait training, better reflecting balance and motor function in real-world walking environments. Therefore, the greater improvements in BBS and FMA-LE scores in the mobile robot group suggest that it may be more effective in improving overall muscle coordination and balance recovery [40].

In this study, the FMA-LE subcomponents of reflex activities and coordination/speed showed statistically significant improvements before and after treatment in both groups, but no significant differences were observed between the groups. This suggests that both robotic systems were effective in improving reflex activities and coordination/speed, indicating positive impacts on these subcomponents regardless of the type of robotic system used. Since reflex activities and coordination/speed are critical for overall balance and gait recovery, these results demonstrate that both robotic training methods contributed to the functional recovery of the patients. The improvement in reflex activity scores reflects the restoration of spinal-level neural reflexes and may indicate recovery of impaired cortical inhibitory control or improved modulation of spinal reflexes. It also suggests the reintegration of lower motor neuron function, which can have a positive impact on the recovery of volitional movements [41,42].

Notably, however, statistically significant improvements in synergies, mixed synergies, and volitional movement were observed only in the experimental group using the mobile robot system. These findings highlight the differential effects of robotic training on various levels of motor recovery. The improvement in “volitional movement within synergies” and “volitional movement mixing synergies” in the mobile group suggests that training in a dynamic, overground environment may promote greater activation of voluntary motor control pathways. While “volitional movement within synergies” reflects the ability to perform voluntary movements constrained within stereotyped flexor or extensor patterns typical of early recovery, “volitional movement mixing synergies” represents a higher level of motor control, involving the ability to combine elements of different movement synergies for more functional tasks [43]. The mobile robot allows for free movement in real-world environments, potentially facilitating a more natural gait retraining process, thus contributing to greater functional recovery in these subcomponents [25,26,30].

When comparing the two groups, no statistically significant differences were found in synergy, mixed synergies, or volitional movement. This suggests that, despite the mobile robot showing significant improvements in specific subcomponents, there was no clear difference between the two groups overall. This highlights the possibility that the effectiveness of robotic training can vary depending on the patient’s individual characteristics and condition. Therefore, personalized rehabilitation plans tailored to the patient’s needs are essential, and careful consideration of the advantages of each robotic system should be part of the treatment strategy [26,30,44]. These findings suggest that while both mobile- and fixed-type robotic gait devices contribute meaningfully to the improvement of gait and balance in stroke patients, mobile robots may offer additional advantages by enabling movement in dynamic, real-world-like environments. This functional difference may be especially beneficial for facilitating more natural gait retraining and enhancing overall sensorimotor integration during rehabilitation [44,45].

The correlation analysis conducted in this study provides important insights into the recovery of gait and balance in stroke patients, particularly regarding the relationships between FAC, BBS, and FMA-LE subcomponents. Notably, the significant correlation between FAC and BBS is consistent with previous research. Other studies have also reported a correlation between FAC and BBS, confirming that walking independence is closely linked to a patient’s balance ability [46,47]. Additionally, the significant correlation observed in this study between BBS and reflex activities suggests that reflex activities play a critical role in balance recovery, which is in line with the findings of Tyson et al. [42]. Their research also highlighted the positive impact of reflex activities recovery on patients’ balance ability, reinforcing the results of this study.

Moreover, the high correlations between the FMA-LE subcomponents of synergy and mixed synergies observed in this study are noteworthy. Kleim et al. emphasized that muscle coordination is a key factor in motor function recovery post-stroke and that the process of restoring coordination through neuroplasticity is crucial [48]. The strong correlation between synergy and mixed synergies in this study supports the idea that the recovery of muscle coordination significantly influences functional recovery, with coordinated recovery among different muscle groups directly contributing to improved balance and motor function [46,47,48].

Additionally, the moderate correlations found in this study between volitional movement, coordination, and speed align with the findings of Langhorne et al. Langhorne’s research indicated that volitional movement tends to recover more slowly in the early stages, while coordination and speed improve more rapidly [48,49]. Similarly, in this study, volitional movement showed a moderate correlation, suggesting that it may recover at a slower pace. In contrast, coordination and speed demonstrated moderate correlations, indicating faster recovery in these areas [48,49,50].

However, there are differences between this study and some previous research. For instance, some research reported that training with a mobile robot resulted in greater improvements in specific subcomponents, particularly volitional movement, while in this study, the mobile robot showed more pronounced improvements in mixed synergies and coordination/speed. This suggests that training outcomes may vary depending on the patient’s condition and the intervention method, highlighting the need for mobile robots to be tailored to the individual physical requirements of the patient [51,52,53].

Although previous studies on the mobile approach supported its effectiveness in overall strengthening and gait correction, this study highlights its particular effectiveness in patients with higher levels of independence, suggesting it as the best approach for improving functional autonomy [26,30].

In this regression analysis, an increase in FAC was significantly associated with an improvement in BBS, indicating that walking independence plays a critical role in balance function. This finding aligns with previous studies, including Tyson et al., which highlighted the close relationship between walking ability and balance performance in stroke patients [42,51,52,53,54].

The regression model also included other lower extremity motor components – such as reflex activities, volitional movements within and without synergies, mixed synergies, and coordination/speed – based on their physiological relevance to balance control. Although only FAC showed a statistically significant effect, including these variables allowed for a more comprehensive exploration of potential influences.

The *R*-squared value of 0.482 suggests a moderate level of explanatory power, supporting the association between FAC and BBS, consistent with Kitaji et al. [55]. The VIF value (1.164) confirmed the absence of multicollinearity, indicating model stability.

While other variables were not statistically significant, their inclusion reveals that FAC has the strongest predictive value for balance recovery. This is consistent with Langhorne et al., who also reported greater influence of walking-related variables on balance outcomes [14,49]. These results underscore the importance of emphasizing walking function in rehabilitation to promote balance recovery. The model’s reliability further supports the need for individualized rehabilitation approaches targeting both ambulation and postural control [55,56].

A convalescent rehabilitation hospital provides intensive treatment for up to 6 months after admission, playing a crucial role in learning and repetition, which are fundamental to rehabilitation [7,8,9]. In this regard, RAGT is also an effective system for repetition and learning. While progressive resistance training, aerobic exercise, CIMT (constraint-induced movement therapy), and task-oriented training are well-known for their effectiveness in rehabilitation, their impact significantly decreases if they are not performed with repetition. Most studies show positive effects immediately after treatment, but it is commonly reported that patients experience a decline in function 3–6 months after treatment ends. Therefore, RAGT may serve as a beneficial therapeutic intervention in convalescent rehabilitation hospitals, helping to establish rehabilitation protocols for intensive early-stage training [51,52,57,58].

This study presents several limitations that may affect the generalizability and reliability of the findings. *A priori* power analysis indicated that a minimum of 102 participants would be required to achieve sufficient statistical power (*α* = 0.05, power = 0.80, Cohen’s *d* = 0.5); however, due to clinical constraints, only 28 participants were enrolled and completed the study. This small sample size weakens the ability to detect subtle group differences, limits statistical power, and increases the influence of individual variability, thereby reducing the generalizability of the results to the broader stroke population.

In addition, robotic gait training was implemented only once per day during the 12-week intervention, as part of a larger rehabilitation program consisting of 14 daily therapy sessions. This limited frequency and duration may not have fully leveraged the potential of robotic therapy, which relies on the principles of repetitive task-specific movement training and neuroplastic adaptation. The insufficient intensity could have attenuated the potential therapeutic gains, especially in patients with more severe impairments.

Another limitation is the lack of differentiation between ischemic and hemorrhagic stroke types. Given their distinct pathophysiological mechanisms and recovery trajectories, the absence of subgroup analysis may obscure potential treatment-specific responses. Furthermore, due to the nature of robotic therapy implementation, only a single-blind design was feasible. This introduces potential bias, as both participant expectations and assessor awareness may have influenced the observed outcomes, reducing the internal validity compared to double-blind trials.

Despite these limitations, the study offers clinically relevant insights. Functional improvements in both balance and lower extremity motor recovery were observed across groups, with slightly superior outcomes in the mobile robot group. Notably, significant correlations between FAC and BBS, and between BBS and reflex activities, underscore the interdependence of walking independence and postural control.

These findings highlight the practical value of robotic-assisted gait training in real-world inpatient rehabilitation settings, where treatment conditions are shaped by staffing, time, and resource constraints. For instance, one participant in the mobile robot group, who initially presented with FAC 1 and high spasticity, demonstrated meaningful gains in gait endurance and voluntary ankle control following structured exposure to dynamic overground training. Such individualized responses reinforce the importance of personalized rehabilitation planning based on patient-specific goals, motor capabilities, and tolerance.

Future studies should include larger sample sizes, stratification by stroke subtype, and longer-term follow-up to validate the sustainability of observed benefits. Moreover, comparative analysis between robotic and traditional therapies, as well as their combined effects, will be essential to optimizing intervention strategies. Ultimately, robotic therapy should be integrated into a patient-centered rehabilitation framework to maximize functional outcomes in diverse stroke populations.

This study investigated and compared the effects of end-effector and mobile robotic gait training on balance, motor, and sensory recovery in stroke patients with low levels of independent ambulation. Both intervention groups demonstrated significant improvements in balance. Notably, the mobile robotic gait training group exhibited slightly greater gains in motor and sensory outcomes. Furthermore, significant correlations were found between FAC and BBS, as well as between BBS and reflex activities, highlighting the strong association between balance and ambulatory independence. These findings support the need for personalized rehabilitation strategies tailored to the patient’s functional status to optimize recovery following stroke.
